# Interleukin-8 is increased in chronic kidney disease in children, but not related to cardiovascular disease

**DOI:** 10.1590/2175-8239-JBN-2020-0225

**Published:** 2021-03-12

**Authors:** Seçil Conkar Tunçay, Eser Doğan, Gülden Hakverdi, Zulal Ülger Tutar, Sevgi Mir

**Affiliations:** 1 Ege University Faculty of Medicine, Department of Pediatric Nephrology, İzmir, Turkey.; 2 Ege University Faculty of Medicine, Department of Pediatric Cardiology, İzmir, Turkey.; 3 Ege University Faculty of Medicine, Department of Department of Biostatistics and Medical Informatics İzmir, Turkey.

**Keywords:** Renal Insufficiency, Chronic, Child, Cardiovascular Diseases, Interleukin-8, Cytokines, Insuficiência Renal Crônica, Criança, Doenças Cardiovasculares, Interleucina-8, Citocinas

## Abstract

**Introduction:**

In this study, we aimed to detect the cytokine that is involved in the early stage of chronic kidney disease and associated with cardiovascular disease.

**Methods:**

We included 50 patients who were diagnosed with predialytic chronic kidney disease and 30 healthy pediatric patients in Ege University Medical Faculty Pediatric Clinic, İzmir/Turkey. Interleukin-8 (IL-8), interleukin-10 (IL-10), interleukin-13 (IL-13), and transforming grow factor-β1 (TGF-β1) levels (pg/mL) were measured by ELISA. Carotid-femoral pulse wave velocity (PWV), augmentation index (Aix), carotid intima media thickness (cIMT), and left ventricular mass index (LVMI) were evaluated as markers of cardiovascular disease. The presence of a cardiovascular disease marker was defined as an abnormality in any of the parameters (cIMT, PWV, Aix, and left ventricular mass index (SVKI)). The patient group was divided into two groups as with and without cardiovascular disease.

**Results:**

Mean Aix and PWV values were higher in CKD patients than controls (Aix: CKD 32.8±11.11%, healthy subjects: 6.74±6.58%, PWV CKD: 7.31±4.34m/s, healthy subjects: 3.42±3.01m/s, respectively; p=0.02, p=0.03). The serum IL-8 levels of CKD were significantly higher than of healthy subjects 568.48±487.35pg/mL, 33.67±47.47pg/mL, respectively (p<0.001). There was no statistically significant difference between IL-8, IL-10, IL-13, TGF-1, in CKD patients with and without cardiovascular disease (p> 0.05).

**Discussion:**

IL-8 is the sole cytokine that increases in pediatric patients with chronic kidney disease among other cytokines (IL-10, IL-13 and TGF-β1). However, we did not show that IL-8 is related to the presence of cardiovascular disease.

## Resumo

**Introdução:**

Neste estudo, o objetivo foi detectar a citocina envolvida no estágio inicial da doença renal crônica e associada à doença cardiovascular.

**Métodos:**

Incluímos 50 pacientes diagnosticados com doença renal crônica pré-dialítica e 30 pacientes pediátricos saudáveis na Clínica Pediátrica da Faculdade de Medicina, Universidade de Ege, İzmir/Turquia. Níveis de interleucina-8 (IL-8), interleucina-10 (IL-10), interleucina-13 (IL-13), fator de transformação do crescimento -β1 (TGF-β1) (pg/mL) foram medidos por ELISA. Velocidade de onda de pulso carotídeo-femoral (VOP), índice de amplificação (AIx), espessura da camada íntima-média da carótida (cIMT), índice de massa do ventrículo esquerdo (IMVE) foram avaliados como marcadores de doença cardiovascular. A presença de marcador de doença cardiovascular foi definida como uma anormalidade em qualquer dos parâmetros (cIMT, VOP, AIx, índice de massa do ventrículo esquerdo (IMVE)). Os pacientes foram divididos em dois grupos como com e sem doença cardiovascular.

**Resultados:**

Valores médios de AIx e VOP foram maiores em pacientes com DRC que nos controles (AIx: DRC: 32,8±11,11%, indivíduos saudáveis: 6,74±6,58%, VOP: DRC: 7,31±4,34m/s, indivíduos saudáveis: 3,42±3,01m/s, respectivamente; p=0,02, p=0,03). Níveis séricos de IL-8 de DRC foram significativamente maiores que de indivíduos saudáveis 568,48±487,35pg/mL, 33,67±47,47pg/mL, respectivamente (p<0,001). Não houve diferença estatisticamente significativa entre IL-8, IL-10, IL-13, TGF-1, em pacientes com DRC com e sem doença cardiovascular (p> 0,05).

**Discussão:**

IL-8 é a única citocina que aumenta em pacientes pediátricos com doença renal crônica entre outras citocinas (IL-10, IL-13 e TGF-β1). Entretanto, IL-8 não se associou à presença de doença cardiovascular.

## Introduction

Compared to healthy children, children with chronic kidney disease (CKD) have shorter life expectancy. Although survival has improved with renal transplantation, cardiovascular disease (CVD) is the most common cause of death in patients with CKD^1^. The incidence of cardiovascular events in children with CKD increases with age and is reported to be 23.9% in the 10-14 age range and 36.9% in children aged 15-19 years^2^. There are traditional and uremia-related risk factors for the development of CVD in CKD. Uremia-related risk factors are dysregulation of the Ca-P-PTH and fetuin-A, treatment-related factors are high dose vitamin D and high dose phosphate binders, and disease-related factors (hypertension). Traditional risk factors are hypertension, obesity, malnutrition, insulin resistance, and dyslipidemia.

Cardiovascular abnormalities begin to develop in early stage CKD. CVD in children is usually asymptomatic in contrast to adults. Left ventricular hypertrophy, increased carotid artery intima media thickness, and vascular calcification are the most common early cardiovascular abnormalities in children with CKD. CKD begins with early vascular changes in children with endothelial damage. Subsequently, vascular calcification develops due to inflammatory response, adhesion molecules, growth factors, and cytokines from endothelial cells. Inflammation in early stage CKD is the main cause of CVD due to endothelial damage^3^. However, the pathogenic mechanism of microinflammation is still unknown. Knowing which cytokines are involved in the pathogenesis of inflammation may allow the development of preventive therapy. Some inflammatory mediators have been shown to be responsible for chronic inflammation in CKD^4^. It is not known which cytokines play a role in the development of CVD in the CKD. We examined whether there was a relationship between CVD and potent proinflammatory and chemotactic cytokines, which are interleukin-8 (IL-8), interleukin-10 (IL-10), interleukin-13 (IL-13), and transforming growth factor-β1 (TGF-β1) in pediatric patients with CKD.

## Material and Method

### Study population, clinical and biochemical characteristics of the patients

This study was carried out between March 2016 and March 2018 in Ege University Faculty of Medicine, Pediatric Clinics, İzmir, Turkey. This single center clinical study enrolled 50 children with CKD and 30 healthy volunteers. Verbal and written consent was obtained from the families and patients. The study was approved by the Clinical Research Ethics Committee of Ege University Faculty of Medicine (16-11/29). Patients with active systemic vasculitis, active infection, renal vascular anomalies, cardiovascular anomalies, and family history of early cardiovascular disease were excluded. None of the patients had active inflammatory conditions or taking immunosuppressive drugs. CKD was defined as persistence of kidney dysfunction for more than 3 months. Children between the ages of 5 and 18 years with estimated glomerular filtration rate (eGFR) between 15 and 60 mL/min/1.73m^2^ and had not started renal replacement therapy were included in the study^5^.

Moreover, we examined 30 healthy volunteers, which were between the same age range, and who went to the clinic for their routine health visits. All measurements and analyses were done to both healthy subjects and pediatric patients with CKD.

eGFR was calculated using the Schwartz formula^6^. Blood plasma was obtained after 12 hours of fasting and stored at -80°C for biochemical tests. Serum urea, creatinine, uric acid, calcium, phosphorus, C-reactive protein (CRP), erythrocyte sedimentation rate (ESR), cystatin C (CysC), glucose (mmol/L), fasting insulin, lipid parameters high density lipoprotein (HDL), low density lipoprotein (LDL), total cholesterol, triglyceride, parathormone (PTH), and homocysteine were detected with an automatic biochemical analyzer (Hitachi 7600; Tokyo, Japan). Age, sex, etiology, weight, height, body mass index (BMI), and systolic and diastolic blood pressure were measured.

### Evaluation of cardiovascular parameters

Arterial stiffness evaluation, carotid-femoral PWV (cfPWV), and augmentation index (Aix) measurements were carried out with a Vicorder (Skidmore Medical Limited, Bristol, UK) device. The peripheral and central arterial pulse wave forms from radial and carotid arteries were recorded with a Vicorder device. For the measurement of arterial stiffness, the patients fasted for 12 hours, rested comfortably in the supine position for 30 minutes, and withhold all antihypertensive drugs prior to PWV and AIx measurement. Mean values of the compound radial waveforms were calculated using the computer program prepared solely for this study. The Aix was calculated as the difference between first and second systolic peaks of the central aortic waveforms, and expressed as the percentage of pulsation length. Echocardiography evaluations were performed by the same pediatric cardiologist using two-dimensional M-mode echocardiography with a 3.5-MHz transducer (HP SONOS 1000 System, Philips, Best, The Netherlands). Measurements consisted of interventricular septal thickness, posterior wall thickness, left ventricular (LV) diameter at end diastole, and LV diameter at end systole. The LV mass was calculated using the formula validated by Devereux and Reichek^7^. Carotid artery ultrasonography was performed to measure carotid intima-media thickness (cIMT) according to a previously described method by an experienced pediatric cardiologist^8^. Measurements were done 3 times and mean values were recorded.

### Measurement of cytokines

IL-8, IL-10, IL-13, and TGF-β1 levels were assayed with high-sensitive ELISA method. Patients with CKD were divided into two groups according to the presence of CVD.

Patients without CVD were classified as group 1, and those with CVD, as group 2, based on reference values of aortic pulse wave velocity set by Aix Reusz et al. for children^9^. The cIMT norms set by Doyon A et al. were used 10. PWV normal values for children according to age and sex determined by Thurn D et al were used^11^. LVH is defined as LV mass >51 g/m^2.7^ or LV mass >115 g per body surface area (BSA) for boys and LV mass >95 g/BSA for girls^12^. The presence of CVD was defined as anomaly in any of cIMT, PWV, Aix, and left ventricular mass index (SVKI); 25 patients had CVD and 25 patients did not.

### Statistical analyses

The SPSS software (Statistical Package for the Social Sciences, version 25.0, IBM Corp, New York, NY, USA) was used for analyses. Numeric variables' suitability to the normal distribution was analyzed with Shapiro-Wilk (n<50) and Kolmogorv-Smirnov (n>=50) tests. Variables are presented as mean ± standard error. Categorical variables are presented as numbers and percentages. Chi-square test and Pearson correlation test were used. P value less than 0.05 was accepted as significance level for all hypotheses.

## Results

Fifty patients with predialytic CKD and 30 healthy children were enrolled in this study. Twenty-seven (54%) of the patients were male and 23 (46%) were female. Seventeen (57%) of the healthy children were female and 13 (43%) were male. The mean age of the patients with CKD was 12.59±4.53 years and of healthy subjects, 13.21±6.02 years. The cause of CKD was reflux/urinary tract infection (n=25), obstructive uropathy (n=3), polycystic kidney disease (n=3), hereditary nephritis (n=3), aplasia/hypoplasia (n=1), metabolic disease (n=2), primary glomerulonephritis (n=1), and unknown (n=12). Of all individuals included in the study, 6 (12%) were at stage 2, 20 (40%) at stage 3, and 24 (48%) at stage 4 CKD. The mean BMI was 19.42±5.12 kg/m^2^ in patients and 20.27±3.12 in healthy subjects. Patients with CKD had lower eGFR than healthy subjects: 32.52±21.42 mL/min/1.73m^2^, 121.51±21.42 mL/min/1.73m^2^, respectively, (p=0.001). The mean duration of CKD was 3.64±5.23 years. The SPB and DBP (mean ± SD) of the patients was 119.40±11.03 and 72.40±16.59 mmHg, respectively. All healthy subjects and patients were normotensive. Total cholesterol and triglycerides were significantly higher in the patient group, while HDL was significantly lower (p=0.09, p=0.08, p=0.09) respectively. There was no difference in demographic characteristics between the two groups (p >0.05) (Table 1).

**Table 1:** Comparison of demographic and clinical data of pediatric patients with ckd and healthy subjects

Variable	CKD patients (n=50)	Healthy subjects (n=30)	p-value
Age (years)	12.59±4.53	13.21±6.02	0.37
BMI (kg/m^2^)	19.63±4.94	20.27±3.12	0.47
SBP (mmHg)	119.40±11.03	109.76±13.89	0.16
DBP (mmHg)	72.40±16.59	71.21±10.41	0.23
CRP (mg/dL)	0.34±0.43	0.11±0.73	0.45
ESR (mm/h)	36.61±35.63	34.21±39.42	0.71
Total cholesterol (mg/dL)	180.20±24.14	210.82±147.31	0.09
Triglycerides (mg/dL)	112.40±37.07	104.27±43.58	0.08
HDL cholesterol (mg/dL)	51.56±11.83	44.40±4.34	0.09
LDL cholesterol (mg/dL)	118.33±15.72	98.75±37.96	0.32
Homocysteine (µmol/L)	17.01±3.12	10.25 ± 6.01	0.69
eGFR (mL/min/1.73m^2^)	32.52±21.42	121.51±21.42	0.001

Mean±SD, CKD: Chronic kidney disease, BMI: Body mass index, SBP: systolic blood pressure, DBP: diastolic blood pressure, CRP: C- reaktif protein, ESR: Erythrocyte sedimentation rate, HDL: High Density Lipoprotein, LDL: Low Density Lipoprotein, eGFR: Estimated glomerular filtration rate.

There was no statistically significant difference between the two groups in hemoglobin, serum Ca, P, and CaxP levels (p> 0.05). PTH was found to be higher in CKD patients than in healthy subjects (p=0.001) (Table 2).

**Table 2:** Comparison of uremia-related cardiovascular disease risk factors between ckd patient and healthy subjects

Variable	CKD patients (n=50)	Healthy subjects (n=30)	P-value
Hemoglobin (g/dL)	12.95 ± 1.46	12.05 ± 1.51	0.45
Serum Ca (mg/dL)	9.32± 0.41	9.32 ± 0.94	0.37
Serum P (mg/dL)	4.42 ±0.86	4.14± 2.83	0.16
Serum Ca x P product (mg^2^/dL^2^)	41.26±7.34	35.71± 24.98	0.34
Serum PTH (pg/mL)	118.3 ± 225.45	34.51 ±7.71	0.001

Mean±SD, CKD: Chronic kidney disease, Ca: calcium, P: phosphorus, PTH: parathormone

Mean Aix and PWV values were higher in CKD patients than in healthy subjects (Aix CKD 32.81±11.11%, healthy subjects 6.74±6.58%, PWV CKD 7.31±4.34m/s, healthy subjects 3.42±3.01m/s) (p=0.02, p=0.03). The serum IL-8 levels of CKD were significantly higher than of healthy subjects (568.48±487.35 pg/mL vs. 33.67± 47.47 pg/mL, p <0.001). IL-10, IL-13, and TGF-β1 levels were not different between CKD and healthy subjects (p> 0.05) (Table 3).

**Table 3:** Cardiovascular disease markers and immunological cytokines in patient and healthy subjects

Parameter	CKD patients	Healthy subjects	p-value
SVKI g/m^2.7^	22.23±7.86	20.01±9.19	0.15
PWV (m/s)	7.31±4.34	3.42±3.01	0.03
cIMT (mm)	0.48±0.063	0.44±0.08	0.63
Aix (%)	32.81±11.11	6.74 ± 6.58	0.02
IL-10 (pg/mL)	15.84±58.245	4.27±10.181	0.28
IL-13 (pg/mL)	4.25±9.55	10.39±36.41	0.11
IL-8 (pg/mL)	568.48±487.35	33.67±47.47	0.001
TGF-β1(pg/mL)	59.93±149.53	65.59±182.23	0.35

Mean±SD, CKD: Chronic kidney disease, SVKI: left ventricular mass index, cIMT: carotid intima media thickness, Aix: augmentation index, PWV: pulse wave velocity, TGF-β1: transforming grow factor -β1, IL-8: interleukin-8, IL-10: interleukin-10, IL-13: interleukin-13. Values are expressed by mean ± SD.

Abnormalities of CVD markers (anomaly in any of cIMT, PWV, Aix and SVKI) were detected in 25 of the 50 CKD patients. There was no statistically significant difference between IL-10, IL-13, IL-8, and TGF-β1 levels in patients with and without CVD (Table 4).

**Table 4:** Clinical and laboratory data for ckd patients with or without cardiovascular disease

	CKD Group 1 Patients (n=25)	CKD Group 2 +CVD Patients (n=25)	p-value
IL-10 (pg/mL)	5.68±10.12	26.01± 81.29	0.55
IL-13 (pg/mL)	5.82± 12.63	2.69±4.65	0.44
IL-8 (pg/mL)	498.24±446.51	638.72±524.67	0.39
TGF-β1(pg/mL)	65.95±150.69	53.91±151.22	0.49

Values are expressed by mean ± SD. Group 1: CKD patients without cardiovascular disease. Group 2: CKD patients with cardiovascular disease. TGF-β1: transforming grow factor -β1, IL-8: interleukin-8, IL-10: interleukin-10, IL-13: interleukin-13.

There was no correlation among IL-8 and inflammation markers (ESR, CRP) with biochemical parameters and eGFR (p>0.005).

## Discussion

Although life expectancy decreases in patients with CKD, CVD remains the leading cause of death. CVD begins in the early stage of CKD due to endothelial damage^13^^,^ 14. In the early stages of CKD, traditional risk factors for CVD development are influenced by inflammation and uremic toxin, while dialysis and drugs are effective in the late stages^15^. Knowledge of the cytokine involved in chronic inflammation in the early stage of CVD formation is important for treatment.

We examined the release of IL-8, IL-10, IL-13, and TGF-β1, potent proinflammatory and chemotactic cytokines, and their prognostic significance in predicting CVD in children with CKD. The reason we chose these specific cytokines in this study is that they are considered to be effective in inflammation underlying atherosclerosis. Previous studies showed that cytokine IL-13 may prevent progression of atherosclerosis^16^. It is thought to suppress inflammation through the production of anti-inflammatory mediators such as IL -10 and TGF-β1^14^. IL-8 was first characterized in 1987. Since then, knowledge regarding its function in leucocyte trafficking and activation has advanced rapidly, especially regarding its role in atherosclerosis. Several studies have identified IL-8 in sites of vascular injury, whereas others have demonstrated that IL-8 potentially plays a role in various stages of atherosclerosis^17^. None of these cytokines have been shown to be associated with reduced glomerular filtration rate^18^.

PWV and Aix increase in end-stage renal disease (ESRD) in childhood. These anomalies have been accepted as markers of the early, asymptomatic phase of the cardiovascular process^19^.

In our study, PWV and Aix anomalies were found in children with CKD. This study demonstrated that CVD develops at an early stage because of the presence of these anomalies in the case of moderate renal failure. The increase in PWV and Aix in CKD children without cIMT and SVKI elevations suggests that there are functional changes before structural changes. The mechanism of formation of arterial stiffness in CKD is unclear. Arterial stiffness is associated with dyslipidemia or hypertension^20^, and is considered to be a sign of CVD onset.

IL-8 is important in the regulation of the acute inflammatory response^21^. IL-8 level was higher in children with CKD, although there was no increase in inflammation markers (CRP, ESR). In our study, none of the patients had any condition to cause acute inflammation. Despite this, IL-8 was elevated in the patient group. Therefore, we think that IL-8 is not only an indicator of acute inflammation but it also increases in chronic inflammation.

IL-8 is one of the cytokines involved in the pathogenesis of CKD^22^. IL-8 has been shown to induce endothelial cells (ECs) dysfunction and proliferation of vascular smooth muscles cells (VSMCs) by stimulating the development of vascular calcification through other risk factors. IL-8 has not been shown to be associated with atherosclerosis and coronary heart disease in adult patients, but has been reported to be associated with overall mortality^23^. Panichi et al. evaluated the effect of serum IL-8 on ESRD patients. IL-8 has been shown to be a strong indicator of CVD and overall mortality in ESRD patients^24^. The first stage of atherosclerosis is endothelial dysfunction. Indicators of endothelial dysfunction are PWV and Aix alteration. Although IL-8 is thought to be a cytokine that may be effective at the stage of endothelial dysfunction, we could not show it. IL-8 was high in the CKD group, and PWV and Aix, which are indicators of endothelial dysfunction, were impaired, but this was not associated with IL-8.

IL-8 can be a marker of inflammation in CKD, but we did not find any relationship between IL-8 levels and CVD risk factors. IL-8 is not an indicator of CVD or endothelial damage in CKD.

Because the onset of CVD in CKD patients at the early stage is characterized by endothelial dysfunction, in this study, we aimed to find the cytokine involved in endothelial dysfunction and to shed light on the treatment using the effective cytokine antagonists.

In our study, PWV, Aix, and IL-8 levels were significantly increased in CKD compared to the healthy subjects. It was shown that cardiovascular changes started at the earliest stages of CKD by means of endothelial dysfunction. Although IL-8 was thought to be the cytokine involved in inflammation in CKD, it was not associated with endothelial dysfunctions in our study.

Limitation of this study is that the role of these specific cytokines cannot be fully explained. Cardiovascular drugs such as ACE / ARB and statins have been shown to have a pleiotropic anti-inflammatory effect, such as inhibition of cytokine production *in vitro*, but these drugs were not evaluate and this is a limitation of our study.

## Conclusion

We found that serum IL-8 levels of CKD were significantly higher than of healthy subjects but there was no significant difference between IL-8 levels in patients with and without CVD. Elevated levels of IL-8 may not be considered as a marker for cardiovascular disease, but probably indicate disease-related inflammation. IL-8 may be a cytokine that can increase CKD, but it cannot be a marker of CVD.

## References

[1] Parekh RS, Carroll CE, Wolfe RA, Port FK (2002). Cardiovascular mortality in children and young adults with
end-stage kidney disease. J Pediatr.

[2] Chavers BM, Li S, Collins AJ, Herzog CA (2002). Cardiovascular disease in pediatric chronic dialysis
patients. Kidney Int.

[3] Liu WC, Zheng CM, Lu CL, Lin YF, Shyu JF, Wu CC (2015). Vitamin D and immune function in chronic kidney
disease. Clin Chim Acta.

[4] Mihai S, Codrici E, Popescu ID, Enciu AM, Albulescu L, Necula LG (2018). Inflammation-Related Mechanisms in Chronic Kidney Disease
Prediction, Progression, and Outcome. J Immunol Res.

[5] Inker LA, Astor BC, Fox CH, Isakova T, Lash JP, Peralta CA (2014). KDOQI US commentary on the 2012 KDIGO clinical practice guideline
for the evaluation and management of CKD. Am J Kidney Dis.

[6] Schwartz GJ, Muñoz A, Schneider MF, Mak RH, Kaskel F, Warady BA (2009). New equations to estimate GFR in children with
CKD. J Am Soc Nephrol.

[7] Devereux RB, Alonso DR, Lutas EM, Gottlieb GJ, Campo E, Sachs I (1986). Echocardiographic assessment of left ventricular hypertrophy:
comparison to necropsy findings. Am J Cardiol.

[8] Touboul PJ, Hennerici MG, Meairs S, Adams H, Amarenco P, Bornstein N (2012). Mannheim carotid intima-media thickness and plaque consensus
(2004-2006-2011). An update on behalf of the advisory board of the 3rd, 4th
and 5th watching the risk symposia, at the 13th, 15th and 20th European
Stroke Conferences, Mannheim, Germany, 2004, Brussels, Belgium, 2006, and
Hamburg, Germany, 2011. Cerebrovasc Dis.

[9] Reusz GS, Shroff R, Kis E, Cseprekal O, Fischer DC, Haffner D (2013). Reference values of aortic pulse wave velocity in a large healthy
population aged between 3 and 18 years. J Hypertens.

[10] Doyon A, Kracht D, Bayazit AK, Deveci M, Duzova A, Krmar RT, 4C Study Consortium (2013). Carotid artery intima-media thickness and distensibility in
children and adolescents: reference values and role of body
dimensions. Hypertension.

[11] Thurn D, Doyon A, Sözeri B, Bayazit AK, Canpolat N, Duzova A, 4C Study Consortium (2015). Aortic Pulse Wave Velocity in Healthy Children and Adolescents:
Reference Values for the Vicorder Device and Modifying
Factors. Am J Hypertens.

[12] Flynn JT, Kaelber DC, Baker-Smith CM, Subcommittee on Screening and Management of High Blood Pressure in
Children (2017). Clinical Practice Guideline for Screening and Management of High
Blood Pressure in Children and Adolescents. Pediatrics.

[13] Bonello L, De Labriolle A, Roy P, Steinberg DH, Okabe T, Pinto Slottow TL (2008). Impact of optimal medical therapy and revascularization on
outcome of patients with chronic kidney disease and on dialysis who
presented with acute coronary syndrome. Am J Cardiol.

[14] Taleb S (2016). Inflammation in atherosclerosis. Archives of cardiovascular diseases.

[15] Baragetti I, El Essawy B, Fiorina P (2017). Targeting Immunity in End-Stage Renal Disease. Am J Nephrol.

[16] Gisterå A, Hansson GK (2017). The immunology of atherosclerosis. Nat Rev Nephrol.

[17] Apostolakis S, Vogiatzi K, Amanatidou V, Spandidos DA (2009). Interleukin 8 and cardiovascular disease. Cardiovasc Res.

[18] Feng YM, Thijs L, Zhang ZY, Yang WY, Huang QF, Wei FF (2018). Glomerular function in relation to circulating adhesion molecules
and inflammation markers in a general population. Nephrol Dial Transplant.

[19] Groothoff JW, Gruppen MP, Offringa M, de Groot E, Stok W, Bos WJ (2002). Increased arterial stiffness in young adults with end-stage renal
disease since childhood. J Am Soc Nephrol.

[20] Jogestrand T, Fehrman-Ekholm I, Angelin B, Berglund L, Gäbel H (1996). Increased prevalence of atherosclerotic wall changes in patients
with hyperlipidaemia after renal transplantation. J Intern Med.

[21] Remick DG (2005). Interleukin-8. Crit Care Med.

[22] Qin Y, Fan F, Zhao Y, Cui Y, Wei X, Kohama K (2013). Recombinant human CXCL8(3-72)K11R/G31P regulates smooth muscle
cell proliferation and migration through blockage of interleukin-8
receptor. IUBMB Life.

[23] Moreno Velásquez I, Gajulapuri A, Leander K, Berglund A, de Faire U, Gigante B (2019). Serum IL8 is not associated with cardiovascular events but with
all-cause mortality. BMC Cardiovasc Disord.

[24] Panichi V, Taccola D, Rizza GM, Consani C, Ghiadoni L, Filippi C (2006). Interleukin-8 is a powerful prognostic predictor of all-cause and
cardiovascular mortality in dialytic patients. Nephron Clin Pract.

